# Supplementary material for: Reindeer Husbandry in Switzerland—Management, Feeding, and Endoparasite Infections
Source: Animals (Basel). 2023 Apr 23;13(9):1444. doi: 10.3390/ani13091444 (PMC10177455; doi:10.3390/ani13091444)
Supplement: Supplementary file 1 [file animals-13-01444-s001.zip › animals-2341725-supplementary.pdf]

animals

MDPI

Appendix A

A. Questionnaire for reindeer herds in Switzerland

A1 Questions about farm/zoo, management and feeding

A1.1 Farm/Zoo

- Since when have you got reindeer?
- How did you become interested in keeping reindeer, what was the reason for it?
- Have you had captive wild animals before?
- What other kind of animals have you got as well?
- Do those species share the enclosure with the reindeer?
- Are the reindeer a hobby or a part of the income?
- How many times per day do you or someone else go see the reindeer? When feeding (once or twice a day), more, less
- Who looks after the animals is it always the same person or does it change?
- Do you have a (personal) veterinary?
- Have you had any kind of difficulties with having reindeer or what were the difficulties/challenges/highlights in the beginning?
- Where do you get your information about reindeer keeping?
  Enclosure
- How many enclosures do you have?
- What height is the fence?
- How big is the enclosure in square meters?
- How is the slope of the enclosure? Flat/steep/in between
- Do you have a rotational pasture or a permanent pasture? If you have a rotational pasture, how is the interval?
- How many animals do you have per enclosure and how is the group separated?
- If you separate the group, what criteria do you use?
- How is the ground in the enclosure? Is there grass, dirt, pavement, gravel, concrete, wood?
- Is there a possibility for the animals to scratch themselves?
- Are there places that give shade on the pasture, like trees? Do the animals prefer those places to the barn?
- Are there other shelters except the barn in the enclosure?
- How is the enclosure exposed to the wind and sun? Is it a northern slope? Is there usually a breeze in the enclosure?
- What altitude is the barn over sea level?
- Are there any wet/moist parts or a creek in the enclosure?
- Can wild animals like foxes get into the enclosure?
- How about cats or dogs?
- Around the enclosure are there other animal pastures or forests? Which?
- Where do you put the excrements of the animals, close by or outside the fence?
- Do you have an extra room for the calves to go to?

A1.2 Barn

- How big is the barn?
- Where is the opening of the barn – south/north?
- How many openings/exits does the Barn have? How high and how wide are they?
- What kind of ground is in the barn? Do you put straw in it?
- How much do the animals use the barn? Do they like to use the barn?

A1.3 Feeding

- What feed do the reindeers (main-feed and supplementary feed) get?
- Hay, Hylage, first or second cut?
- Grass on pasture or do you get grass from outside for them?

Hay, Hylage, first or second cut?
Grass on pasture or do you get grass from outside for them?
Corn
Luzerne – bur clover/alfalfa/lucerne
Special reindeer feed; Producer:
Grain (oats, barley, wheat)
Vegetables:
Apples, other fruits:
Trees, which ones:
Mineral-mix, Salt, Producer
Others:

- Does the feeding differ from one season to another?
- Do you produce the feed yourself?
- Which feed do you buy and where do you get it?
- How many times a day do you feed the animals?
- Are all reindeer able to feed at the same time? Do you have multiple places to feed for the reindeer?
- How much space do you calculate for a reindeer for feeding?
- Is there a roof over the place where you feed them?
- How much feed does one reindeer get? How much would you guess the percentage of hay they eat?
- How do you feed the reindeer? On the ground, hay rack, etc.
- Do you clean the place where they feed regularly and are the leftovers thrown away?
- How much leftovers are the animals allowed to make before you take it out? Percentage?
- Why did you choose this kind of feed?
- Do you seed a special kind of grass in the reindeer pasture?
- Are there trees in the pasture where the reindeer can eat from? Or do you give them other kind of leaves, branches? If yes which kind and how much per week?
- Are there other kind of trees outside the pasture where the animals reach to, what kind?
- Are there any poisonous plants the animals could get?
- How does the pasture look, are there any weeds on it?
- Do you cut the grass in the pasture, or do you put chalk/lime/calcium carbonate on it?
- Do you give the reindeers lichen? What kind? When do the animals get them? Do you collect them or are they bought?
- What kind of treats do your animals get?
- Do you give a mineral supplement? How much do the animals get of it? What products do you use? And how do the animals get it, a stone to lick, in the feed, a powdery kind? Is it always available?
- What kind of ingredients do you think is important in feeding reindeer?
  Watering place:

- How do the animals have access to water? Fountain, automatic drinker, etc.
- Can the water place be heated in Winter?
- Do you have your own water from a well? Is there a creek, river, etc?
- Is there snow in the pasture in winter?

A1.4 Herd management

- Do you breed with your animals?
- Do you have your own breeding male? How often do you change it?
- Have you had incest or the suspicion? If yes, have you had problems with it?
- Do you also buy animals, if yes from where?
- If yes, have you had an idea about the parasite situation from where the animals came? Were the animals quarantined?
- Have you already sold animals? To where?
- Have you done exports or imports? From or to what country?
- Have you also slaughtered animals? If yes, was there anything special noticed in the meat control?
- How is the removal management of the excrements? Is it done regularly? Only the barn or also the pasture? How much is removed?
- Is the dung put back on the pasture?
- Do you put fertilizer on the pasture?
- How often do you clean the barn and how do you do it? With a high-pressure cleaner / chemicals?
- Do you work on a harness with individuals?
- Do you sometimes take some animals out of the enclosure?
- What do you do concerning the claw management? Do you trim them?
- What do you think are difficulties regarding the management in keeping reindeer?

A2 Health situation

- When are your animals sexually mature?
- If you have babies, do they drink only by their own mother or do they also share their mothers?
- Do some of your reindeer show signs of diarrhoea, or have you had problems with diarrhoea, lameness, coughing or acute death in the herd?
- In case you had one or more of these problems already, what was done against it?
- Have you had different problems/diseases?
- In case of death, is the cause of death known? Was a pathological dissection done?
- What do you think are difficulties concerning reindeer health?
- When do your reindeer change the winter fur to summer fur?

A2.1 Parasites

- Have you already had problems with endo- or ectoparasites?
- Do you regularly take fecal samples?
- How often do you deworm the reindeers? Never/once/twice a year/ more often
- When do you deworm the reindeer? Because of a fecal sample result/ because of the season
- How do you deworm the reindeer? Pour-on/per injektionem/over the feed?
- Do you deworm the whole herd or just some animals?
- How do you dose the deworming medicine? Do you guess the weight of the animals, or do you weigh them?
- Why do you deworm in that particular season?
- Which medicine is used for deworming?

Preparation	Active substance	Application
Albex, Valbazen	Albendazol (100mg/ml)	
Cydectin (pour-on, oral, p.inj.)	Moxidectin	
Dectomax (pour-on, p.inj.)	Doramectin	
Eprecis (pour-on, p.inj.), Eprinex, Eprivalan, Eprizero, Neoprinil	Eprinomectin	
Hapadex	Netobiminum	
Noromectin (pour-on, p.inj.), Optimectin (pour-on, p.inj.), Virbamec	Ivermectin	
Panacur	Fenbendazol	
Endex	Levamisol, Triclabendazol	
Closamectin (pour-on)	Closantel, Ivermectin	
Ivomec	Clorsulon, Ivermectin	
Zolvix	Monepantel	

- Do you always use the same deworm medicine or do you change products? In case you change it, what are your reasons for changing?
- Why do you use these medications to deworm the animals, because of the recommendation of the veterinarian?
- Do your animals have problems with insects in summer? Do you use insect repellent on them? Do you have an insect prevention in the barn? If yes, what do you use?
- Do you treat the animals for ectoparasites/insects? If yes, with what?
- How is the behavior of the animals in summer?
